# Comparison of Elecsys and Liaison immunoassays to determine Epstein–Barr virus serological status using further diagnostic approaches to clarify discrepant results

**DOI:** 10.1002/jmv.28166

**Published:** 2022-10-01

**Authors:** Julien Lupo, Mansour Tsougaev, Stéphane Blachier, Guillaume Chovelon, Aurélie Truffot, Corentin Leroy, Joris Giai, Olivier Epaulard, Raphaële Germi, Patrice Morand

**Affiliations:** ^1^ University Grenoble Alpes, Virology Laboratory Grenoble‐Alpes University Hospital Grenoble France; ^2^ Institut de Biologie Structurale (IBS) University Grenoble Alpes, CEA, CNRS Grenoble France; ^3^ Laboratoire BioGroup Saint Martin d'Hères Grenoble France; ^4^ Clinical Investigation Center INSERM CIC1406 Grenoble‐Alpes University Hospital Grenoble France; ^5^ Département de Méthodologie et de l'Information de Santé Grenoble‐Alpes University Hospital Grenoble France; ^6^ Infectious Diseases Unit Grenoble‐Alpes University Hospital Grenoble France; ^7^ Groupe de recherche en infectiologie clinique, CIC‐1406 Inserm—CHUGA—University Grenoble‐Alpes Grenoble France

**Keywords:** Epstein–Barr virus (EBV), immunoassays, serology

## Abstract

Serological markers for Epstein–Barr virus (EBV) infection are commonly used to diagnose infectious mononucleosis and establish a serological status in pretransplant patients. This study prospectively assessed 1043 serum specimens sent to the laboratory for physician‐ordered EBV testing. The three markers—antiviral capsid antigen (VCA) immunoglobulin M (IgM), anti‐VCA immunoglobulin G (IgG), and anti‐Epstein–Barr nuclear antigen (EBNA) antibodies—were tested using the Elecsys and the Liaison immunoassays. Specimens with discrepant results between the two assays were assessed using further EBV diagnostic approaches to conclude on the EBV serological status. In spite of substantial agreement between the two assays (88%) and with the presumed EBV status (>92%), the results showed differences in the performance of the assays. Liaison VCA IgM appeared to be the most sensitive test for the detection of the 38 sera classified as early primary infection in comparison with the Elecsys assay (91.4% vs. 68.6%, *p* = 0.008). Excluding the cases of early primary infection, the sensitivity values of the VCA IgM marker were comparable between the Liaison and Elecsys assays (95.2% and 92.9%, respectively, *p* = 1). Concerning the sera classified as past infection (*n* = 763), the Elecsys assay showed higher sensitivity values for the detection of the VCA and EBNA IgG markers in comparison with the Liaison assay (99.9% and 99.7% vs. 97.4% and 91.2%, respectively, *p* < 0.001). Overall, the Elecsys and Liaison assays showed similar performance. The interpretation of EBV serological profiles based on the clinical context may require serology follow up or further diagnostic approaches in challenging cases.

## 1 INTRODUCTION

Epstein–Barr virus (EBV), also known as human herpes virus 4 (HHV‐4), infects more than 95% of adult individuals and persists over the lifetime in the infected host. Primary infection is usually asymptomatic but can cause infectious mononucleosis (IM), occurring most often in young adults.^1^ EBV is an oncogenic virus associated with epithelial cancers such as nasopharyngeal carcinoma and 10% of gastric cancer cases, as well as lymphomas including Burkitt's lymphoma, Hodgkin's lymphoma, diffuse large B cell lymphoma, natural killer (NK)/T cell lymphoma, and posttransplant lymphoproliferative disorders.^2^ A history of IM has been associated with an increase in the risk of EBV‐positive Hodgkin's lymphoma and multiple sclerosis.^3^, ^4^ Two recent reports supported the evidence that EBV is an etiological factor in multiple sclerosis.^5^, ^6^

EBV serology is mainly used for IM diagnosis and determination of the EBV status in the context of solid organ or hematopoietic stem cell transplantation. Its usefulness has also been explored in EBV‐associated malignancies, in particular in nasopharyngeal carcinoma with the anti‐EBV IgA‐specific antibody marker in combination with serum EBV DNA.^7^ EBV serology testing makes it possible to detect antibodies against different antigens produced at various stages of EBV infection such as viral capsid antigen (VCA), early antigen (EA), and EBV nuclear antigens (EBNA). In clinical practice, three markers are commonly evaluated for the serological diagnosis: anti‐VCA IgM, anti‐VCA (EA) immunoglobulin G (IgG), and anti‐EBNA‐1 IgG antibodies, useful for the determination of EBV serological profiles.^8^ The results of these EBV markers are generally sufficient to conclude in a seronegative status, a primary infection, or a past infection.

Immunofluorescence assays (IFA) is considered the gold standard for EBV serology. Nevertheless, because performing and interpreting IFA is labor‐intensive and sometimes subjective, many laboratories use commercially available immunoassays based on enzyme‐linked immunosorbent assay (ELISA) or chemiluminescent methods. These assays showed good agreement with gold standard assays and are easier to perform. Diagnostic approaches based on IFA, heterophile testing, immunoblot analysis, and polymerase chain reaction (PCR) can be used to clarify some atypical serological results previously determined by immunoassays.^9^

This large prospective study aimed to evaluate the newly developed automated Elecsys EBV immunoassay in comparison with a widely used immunoassay in laboratories, the Liaison EBV immunoassay. Further diagnostic approaches for EBV diagnosis were performed when discrepant results were observed between the two assays. The independent evaluation of automated immunoassays is crucial to help biologists and clinicians in the interpretation of EBV serological status and the management of transplanted patients or patients suspected of IM.

## 2 METHODS

### 2.1 Patients

The patients were included from May to August 2021 during the COVID‐19 pandemic in a city laboratory network (Biogroup) and tested for EBV serology according to the prescription of their general practitioner. Demographic and clinical data were collected if available (symptoms compatible with IM, lymphocytosis, elevated transaminases, and results of CMV, HIV, and toxoplasma serology). All included patients (or legal guardians) were informed and gave their consent to participate in the study in accordance with the ethics committee (CPP Ouest 6‐2020‐A03128‐31).

### 2.2 First‐line assays in the city laboratory

Each patient's serum was tested consecutively for EBV serology with two commercial EBV immunoassays available at the Biogroup City Laboratory, Grenoble, France: the Elecsys EBV assay on the Cobas e801 analyzer (Roche Diagnostics) and the Liaison EBV assay on the Liaison XL analyser (Diasorin). The anti‐VCA IgM, anti‐VCA IgG, and anti‐EBNA IgG antibodies were analyzed with the two automated immunoassays in accordance with the manufacturers' instructions. The results were interpreted according to the cut‐offs described in the manufacturers' instructions (see Supporting Information: Table S1). If the result of one serological marker differed from one assay to another (i.e., a negative vs. positive or equivocal vs. negative/positive result), the serum specimen was sent to the reference laboratory.

### 2.3 Further diagnostic approaches in the reference laboratory

Serum specimens displaying discrepant results between the Elecsys and Liaison assays were evaluated in the reference laboratory (Virology Laboratory). Further diagnostic approaches were performed if the interpretation of the EBV serological pattern differed between the Elecsys and Liaison assays (see Supporting Information: Table S2).

Complementary EBV testing included the Vidas VCA IgM, VCA/EA IgG, and EBNA IgG assays (Biomerieux), anti‐EBV IgM and IgG immunoblot assays (Euroline anti‐EBV Profil 2, Euroimmun France), anti‐VCA IgM and IgG IFA (Euroimmun), heterophile antibody detection (Monospot Latex; Meridian Bioscience), and EBV PCR (EBV R‐gene; Biomérieux).

The Vidas assay was performed for the majority of specimens with discrepant interpretation. If the Vidas assay was not contributive, an IgG or IgM immunoblot assay was performed to rule out the possibility of a false‐positive result in one or several markers. In cases of suspected primary infection not confirmed by previous testing, heterophile antibody testing was performed (considering that this test has a better sensitivity in patients >5 years of age). The IFA was the most frequently initiated to discriminate a primary infection with isolated anti‐VCA IgM antibodies from a seronegative status (with false IgM). EBV DNA detection in serum by PCR associated with isolated anti‐VCA IgM argued in favor of a primary infection. As PCR in serum is positive only 1 or 2 weeks after the onset of symptoms, a negative PCR result could not exclude a diagnosis of primary infection.^10^

### 2.4 Data analysis

The expected EBV status was (i) the status obtained in the city laboratory when the results of the Elecsys and Liaison assays were concordant or (ii) the status established by the reference laboratory when the results of the two assays were discrepant. Serum with isolated anti‐VCA IgM antibodies in the Liaison or Elecsys assays was considered as early primary infection if the diagnosis of primary infection was confirmed by the reference laboratory. EBV status with an indeterminate profile (transition phase, isolated anti‐VCA IgG, or isolated anti‐EBNA IgG) or that remained inconclusive despite the evaluation of the reference laboratory were excluded from the statistical analysis.

Equivocal results obtained with one of the assays were considered as negative for sensitivity calculations and positive for specificity calculations. The sensitivity and the specificity of the different assays were compared using the exact binomial test. The clinical agreement between the Elecsys profiles, the Liaison profiles, and the expected EBV status was established in accordance with the criteria of the respective manufacturers and the following formula using 2 × 2 contingency tables: (True Positive + True Negative)/(True Positive + False Negative + True Negative + False Positive).

## 3 RESULTS

### 3.1 EBV serological profiles characterized in the study

**Figure 1:**
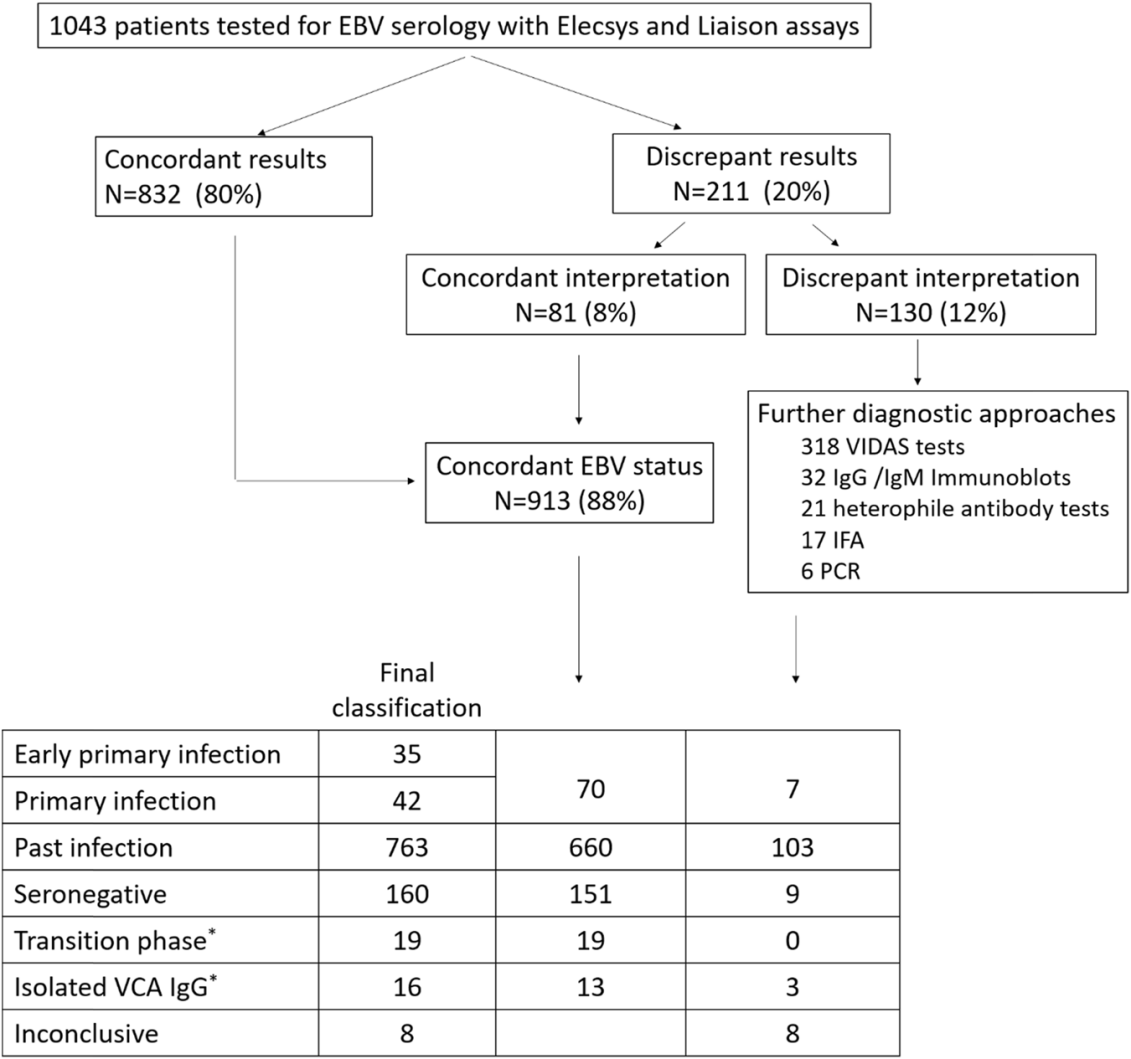
Flowchart of the study. IFA, immunofluorescence assays; IgG, immunoglobulin G; IgM, immunoglobulin M; PCR, polymerase chain reaction; VCA, viral capsid antigen. ^*^Patterns not considered for the evaluation of the assays.

Among the 1043 sera analyzed in both the Elecsys and Liaison assays, 211 (20%) were discordant for at least one marker, but the interpretation of the serological pattern differed from one assay to another for only 130 sera (12%), according to the manufacturers' recommendations (Figure 1). In 80% of cases (*n* = 62) the sera reclassified as concordant displayed a past infection pattern with presence of equivocal anti‐VCA IgM in one assay versus negative IgM antibodies in the other (combined with anti‐VCA and EBNA IgG positive in both assays). In 20% of cases (*n* = 19), the sera displayed an early primary infection pattern in one assay (IgM eq/positive, anti‐VCA IgG and anti‐EBNA negative) and a primary infection profile in the other (IgM eq/positive, anti‐VCA IgG positive, and anti‐EBNA negative). Among the 130 sera requiring assessment at the reference laboratory, a total of 182 complementary analyses were performed including 106 Vidas assays with determination of the three markers (i.e., 318 tests), 32 IgG/IgM immunoblots, 21 tests for detection of heterophile antibodies, 17 IFA for detection of IgG/IgM anti‐VCA antibodies, and six PCR for EBV DNA detection. EBV serological statuses could be attributed to 122 sera out of 130 but the status of eight sera could not be resolved (lack of sample or sera remaining inconclusive despite expert assessment). In conclusion, the overall distribution of serological profiles included the following patterns: *n* = 77 primary infection (7%), *n* = 160 seronegative (16%), *n* = 763 past infection (73%), *n* = 19 transition phase (2%), *n* = 16 isolated anti‐VCA IgG (1%), and *n* = 8 inconclusive (<1%) (Figure 1).

### 3.2 Sensitivity and specificity of the Elecsys and Liaison assays

The sensitivities of the Elecsys and Liaison assays for detection of VCA IgM were calculated using sera classified as early primary infection and primary infection. It should be noted that we considered that equivocal isolated IgM were negative for the calculation of sensitivity, although the sera with this profile are considered as early primary infection according to the interpretation given by the manufacturers' recommendations. The sensitivity value of the Liaison VCA IgM assay was higher than the sensitivity of the VCA IgM Elecsys assay (93.5% vs. 81.8%; *p* = 0.012). Excluding from the calculation the sera classified as early primary infection (isolated anti‐VCA IgM) and considering only primary infection, the sensitivity values between the two assays for the detection of VCA IgM were not statistically different (95.2% for the Liaison assay vs. 92.9% for the Elecsys assay; *p* = 1) (Table 1). Considering the sera classified as past infection or seronegative, the specificity of the Elecsys and Liaison VCA IgM assays were 88.1% and 99.4% versus 91.2% and 97.5%, respectively (*p* = 0.024 and *p* = 0.375, respectively) (Table 2).

**Table 1:** Sensitivity of serological EBV markers determined by the Elecsys and Liaison assays

		Elecsys assay		Liaison assay		
EBV marker	Reference EBV status	% Sensitivity [95% CI]	No. of false negative (equ)	% Sensitivity [95% CI]	No. of false negative (equ)	*p* Value
VCA IgM	Early primary infection (*N* = 35)^a^	68.6 [50.7–83.1]	5 (6)	91.4 [76.9–98.2]	0 (3)	0.008
	Primary infection (*N* = 42)	92.9 [85.1–100.0]	2 (1)	95.2 [88.8–100.0]	0 (2)	1
	Total Primary Infection (*N* = 77)	81.8 [71.4–89.7]	7 (7)	93.5 [85.5–97.9]	0 (5)	0.012
VCA IgG	Total primary infection (*N* = 77)	68.8 [57.3–78.9]	20 (3)	62.3 [50.6–73.1]	32 (NA)	0.332
	Past infection (*N* = 763)	99.8 [99.5–100.0]	1 (0)	97.4 [96.2–98.5]	20 (NA)	<0.001
EBNA IgG	Past infection (*N* = 763)	99.7 [99.4–100.0]	2 (NA)	91.2 [89.5–93.2]	32 (35)	<0.001

*Note*: Equivocal results obtained with one of the assays were considered as negative for sensitivity calculations and positive for specificity calculations.

Abbreviations: CI, confidence interval; EBNA, Epstein–Barr nuclear antigen; EBV, Epstein–Barr virus; equ, equivocal; IgG, immunoglobulin G; IgM, immunoglobulin M; NA, not applicable; VCA, viral capsid antigen.

^a^ Sera with isolated anti‐VCA IgM antibodies in the Liaison or Elecsys assay and classified as primary infection by the reference laboratory.

**Table 2:** Specificity of EBV serological markers determined by the Elecsys and Liaison assays

		Elecsys assay		Liaison assay		
EBV marker	Reference EBV status	% Specificity [95% CI]	N false positive (equ)	% Specificity [95% CI]	N false positive (equ)	*p* Value
VCA IgM	Past infection (*N* = 763)	88.1 [85.8–90.4]	30 (61)	91.2 [89.2–93.2]	24 (43)	0.024
	Seronegative (*N* = 160)	99.4 [98.2–100.0]	0 (1)	97.5 [95.1–99.9]	2 (2)	0.375
VCA IgG	Seronegative (*N* = 160)	97.5 [95.1–99.9]	4 (0)	100.0 [100.0–00.0]	0 (NA)	NA
EBNA IgG	Total primary infection (*N* = 77)	100.0 [95.3–100.0]	0 (NA)	92.5 [83.8–7.1]	0 (6)	NA
	Seronegative (*N* = 160)	100.0 [100.0–100.0]	0 (NA)	98.8 [97.0–100.0]	0 (2)	NA

Abbreviations: CI, confidence interval; EBNA, Epstein–Barr nuclear antigen; EBV, Epstein–Barr virus; equ, equivocal; IgG, immunoglobulin G; IgM, immunoglobulin M; NA, not applicable; VCA, viral capsid antigen.

The sensitivity values for the detection of VCA IgG calculated from sera classified as past infection were higher for the Elecsys assay (99.9% vs. 97.4%; *p* < 0.001) (Table 1), whereas the specificity values calculated from seronegative sera did not differ for the two assays (Table 2). The sensitivity of the Elecsys EBNA IgG assay was significantly higher than that of the Liaison EBNA IgG assay: 99.7% and 91.2%, respectively (*p* < 0.001) (Table 1).

### 3.3 Agreement of Elecsys and Liaison EBV interpretation profile with the expected EBV status

**Figure 2:**
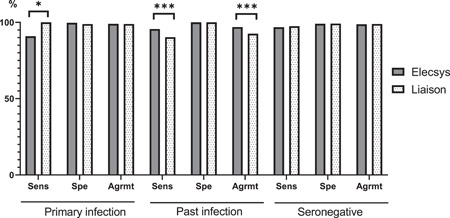
Clinical performance of the Elecsys and Liaison assays. EBV, Epstein–Barr virus; Sens, sensitivity; Spe, specificity; Agrmt, agreement with the reference EBV status. *p < 0.05. ***p < 0.001.

The interpretation of the EBV serological profiles obtained from the Elecsys and Liaison assays was based on the manufacturers' recommendations combining the results of all EBV markers (see Supporting Information: Table S2). Overall, the agreement of the Elecsys and Liaison assays with the expected EBV status was high (>92%), but a number of differences between the two assays were observed (Figure 2). The sensitivity of the Liaison assay for the diagnosis of primary infection was higher than that of the Elecsys assay (100% vs. 90.91%; *p* = 0.016), but the agreement with the EBV expected status was not statistically different between the two assays (*p* = 1). Considering the sera classified as past infection, the agreement with the EBV expected status was higher for the Elecsys assay (96.84% vs. 92.91%; *p* < 0.001) due to its better sensitivity to diagnose past infection (95.67% vs. 90.3%; *p* < 0.001). The details of the differences between the expected EBV status and the EBV serological profiles obtained with the Elecsys and Liaison assays are shown in Tables 3 and 4. With the Liaison assay, 74 of the 763 samples (10%) referenced as past infection were reported as indeterminate or with another status. The most frequently discrepant profile was “isolated VCA IgG” (30/763; 4%) followed by the “transition phase” profile (23/763; 3%) and the “isolated EBNA IgG” profile (15/763; 2%). It is noteworthy that the Liaison instructions do not discriminate the transition phase profile from the primary infection profile when the three EBV markers are present. In comparison, the Elecsys assay reported 33 samples with a misdiagnosis among the 763 samples referenced as past infection. Among them, 30 displayed a transition phase profile. Among the 77 samples classified as primary infection, all were adequately diagnosed by the Liaison assay whereas five serum specimens were reported as seronegative and 2 with isolated VCA IgG by the Elecsys assay. In the 160 sera referenced as seronegative, the Liaison assay reported four cases of primary infection while the Elecsys assay reported four indeterminate profiles with isolated VCA IgG and one primary infection profile.

**Table 3:** Characteristics of discrepant results with the Liaison assay

	Liaison EBV status						
Reference EBV status	EBV seronegative	Primary infection	Past infection	Isolated VCA IgG	Transition phase	Isolated EBNA IgG	Others
EBV seronegative (*N* = 160)	**156**	4	0	0	0	0	0
Primary infection (*N* = 77)	0	**77**	0	0	0	0	0
Past infection (*N* = 763)	3	1	**689**	30	23	15	2^a^

*Note*: Bold values correspond to concordant results between the two assays.

Abbreviations: EBNA, Epstein–Barr nuclear antigen; EBV, Epstein–Barr virus; IgG, immunoglobulin G; IgM, immunoglobulin M; VCA, viral capsid antigen.

^a^ *N* = 2 sera with a anti‐EBNA IgG + anti‐VCA IgM profile.

**Table 4:** Characteristics of discrepant results with the Elecsys assay

	Elecsys EBV status					
Reference EBV status	EBV seronegative	Primary infection	Past infection	Isolated VCA IgG	Transition phase	Isolated EBNA IgG
EBV seronegative (*N* = 160)	**155**	1	0	4	0	0
Primary infection (*N* = 77)	5	**70**	0	2	0	0
Past infection (*N* = 763)	0	0	**730**	2	30	1

*Note*: Bold values correspond to concordant results between the two assays.

Abbreviations: EBNA, Epstein–Barr nuclear antigen; EBV, Epstein–Barr virus; IgG, immunoglobulin G; VCA, viral capsid antigen.

## 4 DISCUSSION

Various versatile new automated assays for EBV serology have launched in the market and need to be analyzed by independent evaluators. To our knowledge, this study is the first independent evaluation of the Elecsys EBV immunoassay in comparison with the Liaison EBV assay. Our prospective study including 1043 patients showed substantial agreement between the Elecsys and Liaison assays, with 88% concordant serological profiles based on the results of the combination of three EBV markers.

The diagnosis of IM is based on clinical manifestations including sore throat, fever, lymphadenopathy, and asthenia, in conjunction with laboratory tests results. Serology remains the method of choice for the diagnosis of IM. In the present study, Elecsys and Liaison assays concluded in the same EBV primary infection profile for 70 sera, whereas 21 sera required further analysis because the results of one assay were suggestive of a primary infection but the other was not. These sera required complementary testing including 63 EBV analysis by Vidas assays, 18 heterophile testing, 13 immunoblot tests, 10 IFA, six PCR, and review of medical records to conclude. Among these 21 discordant results, seven sera were classified as EBV primary infection, five as seronegative, two as isolated VCA IgG, one as past infection, and six remained inconclusive. Among these seven sera newly classified as primary infection, the majority was suspect of early primary infection by the Liaison assay (*n* = 5) or the Vidas assay (*n* = 4) with detection of isolated of VCA IgM antibodies, whereas these sera were classified as seronegative (*n* = 5) or indeterminate (two sera with isolated VCA IgG at a low level) by the Elecsys assay. Of note, for three sera, the VCA IgM index determined by the Elecsys assay was very close to the cutoff (between 0.55 and 0.60).

In case of suspicion of IM and negative results, further analyses or serological follow‐up should be performed so as not to rule out this diagnosis. False negative results could result to unnecessary investigations and delayed or wrong diagnoses. The interpretation of a serological result should also consider the index given by the test. The conjunction of clinical symptoms suggestive of IM and IgM index close to the cut off can suggest an early infection requiring new specimen samples collected few days later to conclude.

Previous reports showed that a transition phase profile (i.e., presence of the three markers) could be related to a primary infection^11^, ^12^ as indicated in the Liaison assay's instructions. This pattern generated a majority of discrepant results between the two assays, which required exploration of 54 sera. Among the 24 sera classified as the transition phase by the Liaison assay, the Elecsys assay concluded in a past infection for 23 sera and a primary infection for one. Among the 30 sera classified as a transition phase by the Elecsys assay, the Liaison assay concluded in a past infection for 27 sera and three sera displayed isolated VCA IgG. Among these 54 sera, 32 sera (60%) were classified as a past infection by the Vidas assay, whereas 22 sera (40%) still displayed a transition phase pattern. Immunoblot IgG tests on these 22 sera clearly showed the presence of EBNA‐1 and the p22 marker, ruling out the possibility of a recent primary infection less than 2 months old.

In the clinical setting, the definitive interpretation of this pattern is often difficult and is based on clinical data (age, stage of illness) and the results of further EBV testing to clearly discriminate the possible end of a primary infection from a past infection with false IgM (cross‐reaction, nonspecific reaction of the immune system) or a serological reactivation profile. Our investigations showed that a transition phase pattern is commonly associated with a past infection. In accordance with our results, Klutts et al.^13^ demonstrated that this profile, upon retrospective clinical review, is also mostly associated with past infection. These results could contrast with previous studies reporting more cases of primary infection with this profile.^11^, ^12^ but we explored only discrepant results of these two assays. Investigation of concordant results harboring this profile may result in increased classification of primary infection cases.

EBV serology is also useful to determine the serological status in the context of transplantation to match the EBV status of the donor with that of the recipient and assess the risk of lymphoproliferative disorders.

In this setting, both assays showed an excellent specificity regarding seronegative status, but the study showed differences in the performance of the assays in clearly ascertaining the status of a past infection. Among the sera classified as past infection, the Liaison assay reported 10% discrepant profiles with the EBV status established by the reference laboratory. The most frequently discrepant profiles obtained with the Liaison assays were isolated IgG VCA (*n* = 30), presence of the three markers (*n* = 23), isolated EBNA IgG (*n* = 15), and seronegative status (*n* = 3). Isolated VCA and EBNA IgG patterns obtained with the Liaison assay were in line with a relative lack of sensitivity of VCA and EBNA IgG markers with this assay.^14^ Previous studies reported that an isolated VCA IgG pattern is commonly associated with a past infection and, to a lesser extent, can also correspond to a primary infection.^11^, ^13^, ^15^, ^16^ Among the discrepant results displaying an isolated IgG VCA IgG profile in the Elecsys assay (*n* = 14), we classified four sera as seronegative, two sera as primary infection, three sera as confirmed isolated VCA IgG (using the immunoblot IgG test), two sera as past infection, and three sera remained inconclusive. All discrepant results with isolated VCA IgG with the Liaison assays were classified as past infection. An isolated EBNA IgG pattern with the Elecsys assay was very uncommon (*n* = 1). This serum classified as past infection by the immunoblot IgG analysis was found seronegative by the Liaison assay and displayed an isolated EBNA IgG pattern with the Vidas assay.

The main risk factors for developing a lymphoproliferative disorder is the development of primary infection in a seronegative patient receiving a transplant from a seropositive donor. Delivering a false seronegative status can be very critical given the increased risk of lymphoproliferative disorders in a seronegative patient receiving the transplant from a nonidentified seropositive donor. In our study, we showed that this risk is very limited using Elecsys and Liaison assays (risk estimated at 0% and 0.4%, respectively). On the other hand, given the scarcity of seronegative donor, a false positive result can deprive a seronegative recipient from an organ essential for its survival. Ours results showed that this risk was relatively rare and similar with the two assays (2.5%–3%).

This study has several limitations. The period of inclusion occurred during the COVID‐19 pandemic in a context of human contact prevention and social distancing measures, which may interfere with the recruitment of primary infection cases.^17^ We strictly followed the cutoffs and the clinical interpretation provided by the manufacturers' recommendations to classify the results of both assays as concordant or discrepant. In some discrepant cases (estimated at <10%), index values given by the assays could be very close to their respective cutoff and we cannot exclude a similar pattern in the two assays in cases of repeated testing (which was not done in the present study). Since we cannot rule out that two immunoassays using similar antigen preparations (i.e., the Liaison and Vidas assays) have the same behavior in antibody testing, we prefer performing several techniques to limit the risk of misclassification on the EBV status rather than reason according to a majority approach. In the absence of a reference method carried out on all samples, we can neglect the possibility that two assays simultaneously presented false results among the 913 concordant results. Finally, we did not evaluate potentially cross‐reactive samples.^18^ Contrary to a recent evaluation,^19^ the strength of our study is its prospective design including 1043 patients who were not selected a priori and the resolution of discrepant results. Moreover, the Liaison and Elecsys assays were performed on fresh samples and samples requiring further EBV testing underwent only one step of freezing/thawing, which limited the risk of pre‐analytical interference, in particular on the VCA IgM marker.

In conclusion, this study showed the similar performance of the Elecsys and the Liaison assays to determine EBV serological status. The Elecsys assay showed an excellent sensitivity for the determination of VCA IgG and EBNA IgG markers in comparison with the Liaison assay. Nevertheless, the lack of sensitivity of the Liaison VCA and EBNA IgG markers is in most cases without clinical significance in a context of organ donation if the two markers are concomitantly performed. The Liaison VCA IgM marker was the most sensitive test for the diagnosis of early primary infection. In case of a suspected IM, serology follow‐up or further EBV testing should be carried out to avoid a false‐negative result of the VCA IgM marker at an early stage of infection. Given the differences in the performance of each single EBV marker in a given commercial assay, the use of more than one automated assay or further diagnostic approaches could be useful to resolve certain difficult profiles.

## AUTHOR CONTRIBUTIONS

Julien Lupo designed the study, analyzed the data and wrote the draft. Mansour Tsougaev performed manual testing, compiled the data and analyzed the data. Guillaume Chovelon and Stéphane Blachier obtained the patient's consent, supervised the Elecsys and Liaison immunoassays and compiled the data. Aurélie Truffot and Olivier Epaulard reviewed the manuscript. Corentin Leroy performed the statistical analysis. Joris Giai performed the statistical analysis and reviewed the manuscript. Raphaële Germi discussed the data and participated in the drafting. Patrice Morand analyzed the data and reviewed the manuscript All authors read and approved the entire content of the final manuscript.

## CONFLICT OF INTEREST

The authors declare no conflict of interest.

## Supporting information

Supplementary information.

## Data Availability

The data that support the findings of this study are available from Roche Diagnostics upon reasonable request.
