# Supplementary material for: Comparison of Elecsys and Liaison immunoassays to determine Epstein–Barr virus serological status using further diagnostic approaches to clarify discrepant results
Source: J Med Virol. 2022 Oct 1;95(1):e28166. doi: 10.1002/jmv.28166 (PMC10092878; doi:10.1002/jmv.28166)
Supplement: Supplementary file 1 — Supplementary information. [file JMV-95-0-s001.docx]

**Supplemental Data Table S1**. Cut-offs of the three immunoassays used in the study

|  | Elecsys | Liaison | Vidas |
| --- | --- | --- | --- |
|  | *index* | *U/mL* | *index* |
| EBV IgM | <0.6: negative  [0.6–1]: equivocal  ≥1: positive | <20: negative  [20–40]: equivocal  ≥40: positive | <0.12: negative  [0.12–0.18]: equivocal  ≥0.19: positive |
| EBV VCA IgG | <0.7: negative  [0.7–1]: equivocal  ≥1: positive | <20: negative  ≥20: positive | <0.10: negative  [0.10–0.20]: equivocal  ≥0.21: positive |
| EBV EBNA IgG | <1: negative  ≥1: positive | <5: negative  [5–20]: equivocal  ≥20: positive | <0.10: negative  [0.10 - 0.21]: equivocal  ≥ 0.21: positive |

**Supplemental Data Table S2**. Interpretation of EBV serological profiles according to manufacturers’ instructions and the reference laboratory

|  |  |  | **EBV serological profiles** | | | |
| --- | --- | --- | --- | --- | --- | --- |
| **VCA IgM** | **VCA IgG*** | **EBNA IgG** | **Elecsys** | **Liaison** | **VIDAS*** | **Reference laboratory** |
| NEG | NEG | NEG | EBV negative | EBV negative | EBV negative | EBV negative |
| NEG | *Eq* | NEG | EBV negative | NA | NA | Indeterminate |
| *Eq* | NEG | NEG | Early PI | Early PI | NA | Early PI |
| **POS** | NEG | NEG | Early PI | Early PI | Early PI | Early PI |
| *Eq* | *Eq* | NEG | PI | PI | NA | PI |
| **POS** | *Eq* | NEG | PI | PI | PI | PI |
| **POS** | **POS** | NEG | PI | PI | PI | PI |
| *Eq* | **POS** | NEG | PI | PI | PI | PI |
| **POS** | **POS** | **POS** | Transient phase | PI or transient phase | Transient phase | Indeterminate |
| **POS** | *Eq* | **POS** | Transient phase | NA | NA | Indeterminate |
| *Eq* | **POS** | **POS** | Past infection | Past infection | NA | Past infection |
| NEG | **POS** | **POS** | Past infection | Past infection | Past infection | Past infection |
| NEG | *Eq* | **POS** | Past infection | NA | NA | Past infection |
| *Eq* | *Eq* | **POS** | Past infection | NA | NA | Past infection |
| NEG | **POS** | NEG | Indeterminate | Indeterminate | Indeterminate | Indeterminate |
| NEG | NEG | **POS** | Indeterminate | Indeterminate | Indeterminate | Indeterminate |
| All other combinations | | | Indeterminate | Indeterminate | Indeterminate | Indeterminate |

*The VIDAS assay explores VCA/EA IgG antibodies.

PI: primary infection; NA: not applicable or not defined by the manufactures’ instructions; NEG: negative; POS: positive; Eq: equivocal
